# Accurate SARS-CoV-2 seroprevalence surveys require robust multi-antigen assays

**DOI:** 10.1038/s41598-021-86035-2

**Published:** 2021-03-23

**Authors:** Christos Fotis, Nikolaos Meimetis, Nikos Tsolakos, Marianna Politou, Karolina Akinosoglou, Vaia Pliaka, Angeliki Minia, Evangelos Terpos, Ioannis P. Trougakos, Andreas Mentis, Markos Marangos, George Panayiotakopoulos, Meletios A. Dimopoulos, Charalampos Gogos, Alexandros Spyridonidis, Leonidas G. Alexopoulos

**Affiliations:** 1 Biomedical Systems Laboratory, National Technical University of Athens, Athens, Greece; 2 ProtATonce Ltd, Demokritos Science Park, Athens, Greece; 3 Department of Clinical Therapeutics, Alexandra General Hospital, National and Kapodistrian University of Athens, Athens, Greece; 4 Division of Infectious Diseases, Department of Internal Medicine, University Hospital of Patras, Patras, Greece; 5 Department of Cell Biology and Biophysics, Faculty of Biology, National and Kapodistrian University of Athens, Athens, Greece; 6 Medicinal Microbiology Laboratory, Hellenic Pasteur Institute, Athens, Greece; 7 Pharmacology Laboratory, University of Patras, Patras, Greece; 8 National Public Health Organization, Athens, Greece; 9 Department of Internal Medicine, BMT Unit and CBMDP Donor Center, University of Patras, Patras, Greece

**Keywords:** Population screening, Epidemiology

## Abstract

There is a plethora of severe acute respiratory syndrome-coronavirus-2 (SARS-CoV-2) serological tests based either on nucleocapsid phosphoprotein (N), S1-subunit of spike glycoprotein (S1) or receptor binding domain (RBD). Although these single-antigen based tests demonstrate high clinical performance, there is growing evidence regarding their limitations in epidemiological serosurveys. To address this, we developed a Luminex-based multiplex immunoassay that detects total antibodies (IgG/IgM/IgA) against the N, S1 and RBD antigens and used it to compare antibody responses in 1225 blood donors across Greece. Seroprevalence based on single-antigen readouts was strongly influenced by both the antigen type and cut-off value and ranged widely [0.8% (95% CI 0.4–1.5%)–7.5% (95% CI 6.0–8.9%)]. A multi-antigen approach requiring partial agreement between RBD and N or S1 readouts (RBD&N|S1 rule) was less affected by cut-off selection, resulting in robust seroprevalence estimation [0.6% (95% CI 0.3–1.1%)–1.2% (95% CI 0.7–2.0%)] and accurate identification of seroconverted individuals.

## Introduction

There is an urgent need for reliable and highly accurate SARS-CoV-2 serological tests for the diagnosis of recent or prior infection and estimation of population-wide seroprevalence^1,2^. More than 300 new SARS-CoV-2 serological tests are currently in development (updated at https://www.finddx.org/covid-19/pipeline). Assessing antibody presence with a single-readout assay is performed by selecting a cut-off value, above which the antibody is considered present (typically, three standard deviations (SD) above the negative mean distribution)^3^. The majority of developed assays report high sensitivity and specificity in clinical samples obtained either from hospitalized SARS-CoV-2-infected patients or before the COVID-19 era^4^. However, there is growing evidence that assays with seemingly good clinical performance might not lead to reliable diagnostic outcomes in low seroprevalence epidemiological studies, where asymptomatic carriers with low antibody titers are overrepresented^2,5^. One approach that holds promise in addressing this issue, is the development of multi-antigen assays coupled with consensus-based rules.

Recently, a number of multi-antigen SARS-CoV-2 serological assays that detect antibodies against multiple antigens have been developed^6–11^. Some of which are already commercially available. Examples include the xMAP^®^ SARS-CoV-2 Multi-Antigen IgG Assay, a bead-based assay developed by Luminex Corp which has received Emergency Use Authorisation by the US Food and Drug Administration and the Sinommune™ Multiplex Antigen Microarray which is based on protein array technology and offers various choices of antigen readouts^12^. Due to their consensus-based approach, multi-antigen assays report increased specificity in clinical samples, without the cost of reduced sensitivity, compared to their single-antigen counterparts and hence provide an overall improved performance. Furthermore, because of the multiple antigen readouts, they can increase the certainty of diagnosis in borderline cases, i.e. low-titer positive samples^13^. To the best of our knowledge, how this enhanced clinical performance and robustness of multi-antigen approaches translates to low seroprevalence epidemiological studies has yet to be experimentally investigated.

In this study, we developed a bead-based multiplex serological assay for the simultaneous detection of antibody response against the N, S1 and RBD SARS-CoV-2 antigens and applied it to a low seroprevalence epidemiological study. The multi-antigen approach requiring partial agreement between single-antigens (RBD&N|S1 rule) was first evaluated in well-defined clinical samples and validated against commercially available single-antigen assays, showing improved performance. In order to experimentally elucidate how this increase in performance transfers to an epidemiological setting, we compared antibody responses against the N, S1 and RBD antigens in 1255 blood donors across Greece (26% female; median age, 42 years; range 17–65). We report striking differences in seroconversion calls between the different antigens that resulted in a wide range of estimated seroprevalence rates and conflicting diagnoses of positive individuals. On the other hand, the multi-antigen RBD&N|S1 approach resulted in a more robust estimate of seroprevalence and accurate identification of seroconverted individuals.

## Results

### Clinical performance of SARS-CoV-2 multiplex assay

The clinical performance of the developed multiplex assay was evaluated in 155 serum samples from 77 PCR-confirmed COVID-19 cases and 78 pre-pandemic individuals (Table 1). The sensitivity and specificity of the assay were calculated for the three single antigen readouts and for 11 multi-antigen consensus-based rules for detection of total, IgG, IgA and IgM isotypes. Performance was evaluated using a cut-off value of mean plus three standard deviation (SD) for each antigen and the results are presented in Table 2. When assessed individually, antigens (RBD, N and S1) were equally specific producing one or two false positives out of the 78 negative samples tested [specificity 97.4% (95% CI 91.1–99.3%) to 98.7% (95% CI 92.7–99.8%)]. Rules-based approaches, requiring at least two of the antigens to be above the cut-off (i.e. rules using an AND gate between two antigens) for reporting a positive result, improved assay specificity in all isotypes *vs*. individual antigens (Table 2). On the other hand, rules, requiring at least one/any of the antigens to be above the cut-off (i.e. OR-based rules), did not improve assay performance when compared to the best performing individual antigen for the respective antibody isotype. Overall, rules that utilized all three antigens and set a positive result when at least two of them were above the cut-off (Antigen A AND [B OR C] reported as A&B|C) showed improved performance, which was further investigated in terms of its statistical significance. On this front, the power of the statistical tests, comparing the sensitivity and specificity between the single-antigen (RBD, N and S1) and multi-antigen rules for the detection of total antibody, was calculated and is presented in Table 3. In the comparison of specificity, the calculated power (0.816) indicates that there is a high chance that the observed improvement in specificity of the multi-antigen rules is correctly classified as statistically significant (Table 3). On the other hand, the power calculation shows that no statistically significant differences can be identified between the sensitivities of the single-antigen tests and multi-antigen rules, given the sample size of 77 PCR-confirmed COVID-19 cases. Similar results were obtained when different cut-off values, based on ROC analysis, were used (see Supplementary Table S7, Supplementary Fig. S4). A detailed analysis of antibody response for the clinical samples, along with a power calculation detailing the ability of the test to discriminate between positive and negative cases, is presented in Supplementary Material S1.3.

**Table 1:** Samples used throughout the study.

Cohort type	Sample type	Sample source	No of samples	Demographics		
				Median age (range)	Gender	Days post infection (range)
Clinical	Positive serum (SARS-CoV-2 PCR-confirmed cases)	Alexandra General Hospital, Athens	60	49 (20–74)	65% M 35% F	46 (13–87)
		University Hospital of Patras	17			
	Negative serum (banked samples from 2018 to 2019)	University Hospital of Patras	78	57 (17–71)	51% M 49% F	N/A
Population screen	Serum from blood donors	Multi-center/University of Patras	1225	42 (17–65)	74% Μ 26% F	N/A

**Table 2:** Clinical sensitivity and specificity of single-antigen assays and multi-antigen rules.

Rule	Description	Total (IgG/IgM/IgA)		IgG		IgA		IgM	
		Sensitivity [95% CI]	Specificity [95% CI]	Sensitivity [95% CI]	Specificity [95% CI]	Sensitivity [95% CI]	Specificity [95% CI]	Sensitivity [95% CI]	Specificity [95% CI]
N	Positive for N	0.948 [0.874, 0.980]	0.974 [0.911, 0.993]	0.948 [0.874, 0.980]	0.974 [0.911, 0.993]	0.519 [0.410, 0.627]	0.987 [0.927, 0.998]	0.221 [0.143, 0.325]	0.974 [0.911, 0.993]
S1	Positive for S1	0.961 [0.892, 0.987]	0.974 [0.911, 0.993]	0.948 [0.874, 0.980]	0.987 [0.927, 0.998]	0.377 [0.277, 0.488]	0.974 [0.911, 0.993]	0.831 [0.732, 0.899]	0.974 [0.911, 0.993]
RBD	Positive for RBD	**0.987** [0.930, 0.998]	0.974 [0.911, 0.993]	**0.974** [0.910, 0.993]	0.987 [0.927, 0.998]	0.935 [0.857, 0.972]	0.974 [0.911, 0.993]	0.792 [0.689, 0.868]	0.974 [0.911, 0.993]
N & S1	Positive for N and S1	0.935 [0.857, 0.972]	**1.000** [0.953, 1.000]	0.935 [0.857, 0.972]	**1.000** [0.953, 1.000]	0.247 [0.164, 0.354]	0.987 [0.927, 0.998]	0.208 [0.132, 0.311]	**1.000** [0.953, 1.000]
RBD & S1	Positive for RBD and S1	0.961 [0.892, 0.987]	**1.000** [0.953, 1.000]	0.948 [0.874, 0.980]	**1.000** [0.953, 1.000]	0.377 [0.277, 0.488]	**1.000** [0.953, 1.000]	0.779 [0.675, 0.857]	0.987 [0.927, 0.998]
RBD & N	Positive for N and RBD	0.948 [0.874, 0.980]	**1.000** [0.953, 1.000]	0.948 [0.874, 0.980]	0.987 [0.927, 0.998]	0.506 [0.397, 0.615]	**1.000** [0.953, 1.000]	0.208 [0.132, 0.311]	**1.000** [0.953, 1.000]
RBD&N|S1	Positive for RBD and N or S1	0.974 [0.910, 0.993]	**1.000** [0.953, 1.000]	0.961 [0.892, 0.987]	0.987 [0.927, 0.998]	0.636 [0.525, 0.735]	**1.000** [0.953, 1.000]	0.779 [0.675, 0.857]	0.987 [0.927, 0.998]
N & RBD|S1	Positive for N and RBD or S1	0.948 [0.874, 0.980]	**1.000** [0.953, 1.000]	0.948 [0.874, 0.980]	0.987 [0.927, 0.998]	0.506 [0.397, 0.615]	0.987 [0.927, 0.998]	0.208 [0.132, 0.311]	**1.000** [0.953, 1.000]
S1 & RBD|N	Positive for S1 and RBD or N	0.961 [0.892, 0.987]	**1.000** [0.953, 1.000]	0.948 [0.874, 0.980]	**1.000** [0.953, 1.000]	0.377 [0.277, 0.488]	0.987 [0.927, 0.998]	0.779 [0.675, 0.857]	0.987 [0.927, 0.998]
RBD|N&S1	Positive for RBD or N and S1	**0.987** [0.930, 0.998]	0.974 [0.911, 0.993]	**0.974** [0.910, 0.993]	0.987 [0.927, 0.998]	0.935 [0.857, 0.972]	0.962 [0.893, 0.987]	0.792 [0.689, 0.868]	0.974 [0.911, 0.993]
S1|RBD&N	Positive for S1 or RBD and N	0.974 [0.910, 0.993]	0.974 [0.911, 0.993]	0.961 [0.892, 0.987]	0.974 [0.911, 0.993]	0.636 [0.525, 0.735]	0.974 [0.911, 0.993]	0.831 [0.732, 0.899]	0.974 [0.911, 0.993]
N|RBD&S1	Positive for N or RBD and S1	0.974 [0.910, 0.993]	0.974 [0.911, 0.993]	0.961 [0.892, 0.987]	0.974 [0.911, 0.993]	0.649 [0.538, 0.747]	0.987 [0.927, 0.998]	0.792 [0.689, 0.868]	0.962 [0.893, 0.987]
RBD & S1 & N	Positive for all three	0.935 [0.857, 0.972]	**1.000** [0.953, 1.000]	0.935 [0.857, 0.972]	**1.000** [0.953, 1.000]	0.247 [0.164, 0.354]	**1.000** [0.953, 1.000]	0.208 [0.132, 0.311]	**1.000** [0.953, 1.000]
RBD|N|S1	Positive for any	**0.987** [0.930, 0.998]	0.923 [0.842, 0.964]	**0.974** [0.910, 0.993]	0.962 [0.893, 0.987]	**0.948** [0.874, 0.980]	0.948 [0.875, 0.980]	**0.857** [0.762, 0.918]	0.936 [0.856, 0.972]

The highest values of each column are highlighted in bold.

**Table 3:** Power calculations for the comparison of specificity and sensitivity between single-antigen tests and rules for total antibody detection.

Rule	Specificity			Sensitivity		
	RBD	N	S1	RBD	N	S1
RBD & N|S1	0.816	0.816	0.816	0.133	0.223	0.099
N & RBD|S1	0.816	0.816	0.816	0.529	0.050	0.085
S1 & RBD|N	0.816	0.816	0.816	0.317	0.085	0.050
RBD & N & S1	0.816	0.816	0.816	0.133	0.223	0.099

**Figure 1:**
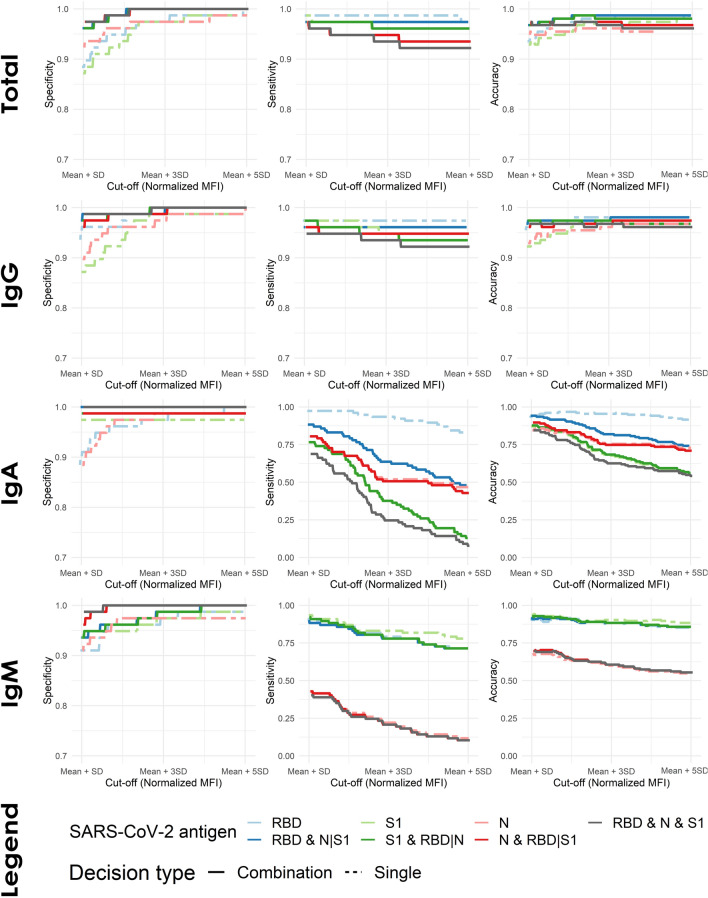
Assay performance metrics for different cut-off thresholds. For each antibody isotype, sensitivity, specificity, and accuracy values were calculated for increasing cut-off thresholds based on the distribution of each antigen’s negative samples. The values on the x axis are normalized MFI, while the different performance metrics are presented as ratios. The single antigen tests are presented with dashed lines, while the multi-antigen rules as solid lines.

To assess the robustness of the multi-antigen rules their performance was evaluated for a wide range of cut-off values. Sensitivity, specificity and accuracy were calculated for gradually increasing threshold (cut-off) values, based on the negative sample distribution of each antigen and antibody isotype and are presented in Fig. 1. Rules using all antigens and requiring two or all three to be higher than the threshold were included in the analysis. Across all isotypes, rules exhibited a more robust profile and were less affected by changes in the cut-off thresholds compared to individual antigens. Rules provided a clear benefit in assay specificity with specificities of 100% (95% CI 95.3–100%) achieved at much lower cut-offs compared to individual antigens. Additionally, for total and IgG antibody detection, assay sensitivity was retained at high levels across a wide range of cut-offs resulting in an overall more robust and accurate assay. The statistical significance of the robustness in sensitivity and specificity of the single-antigen assays and multi-antigen rules was evaluated using the McNemar’s test. The McNemar’s test was applied on each assay to test for differences in specificity and sensitivity when different cut-off values, based on the distribution of negative samples, are considered. The resulting p-values of all comparisons are presented in Table 4. With a 0.05 significance threshold, the p-values of Table 4 show that each single-antigen test (RBD, N and S1) exhibits a statistically significant difference in specificity for two out of three comparisons between cut-off values. On the other hand, the multi-antigen rules show no difference in terms of specificity across all comparisons, indicating a significant improvement in robustness over the single-antigen tests. In terms of sensitivity, both the single-antigen tests and multi-antigen rules show no significant difference across all comparisons, indicating that they are able to maintain a robust sensitivity profile for a wide range of cut-off values. The only exception is the RBD&N&S1 rule which shows a significant difference in sensitivity when the Mean + SD and Mean + 5 SD cut-off values are used. Overall, the results in Tables 2, 3, 4 and Fig. 1 indicate that the RBD&N|S1 rule exhibits a significant improvement in robustness and specificity, compared to all other single-antigen test and rules, while at the same time retaining very high sensitivity.

**Table 4:** p-values of the McNemar’s test to compare the performance of each rule across different cut-off values for total antibody detection.

Rule	Specificity			Sensitivity		
	Mean + SD vs Mean + 5SD*	Mean + SD vs Mean + 3SD*	Mean + 3SD vs Mean + 5SD*	Mean + SD vs Mean + 5SD*	Mean + SD vs Mean + 3SD*	Mean + 3SD vs Mean + 5SD*
N	**0.0253**	**0.0455**	0.317	0.0833	0.157	0.317
S1	**0.00157**	**0.00468**	0.157	0.157	0.157	NA
RBD	**0.0027**	**0.00815**	0.157	0.317	NA	0.317
RBD & N|S1	0.0833	0.0833	NA**	NA	NA	NA
N & RBD|S1	0.157	0.157	NA	0.0833	0.157	0.317
S1 & RBD|N	0.0833	0.0833	NA	0.317	0.317	NA
RBD & S1 & N	0.157	0.157	NA	**0.0455**	0.0833	0.317

p-values lower than the 0.05 threshold are highlighted in bold.

*Mean and standard deviation (SD) of each antigen’s negative sample distribution.

**The McNemar’s test is Not Applicable (NA) because no differences exist.

### Assay validation against commercial antibody tests

**Figure 2:**
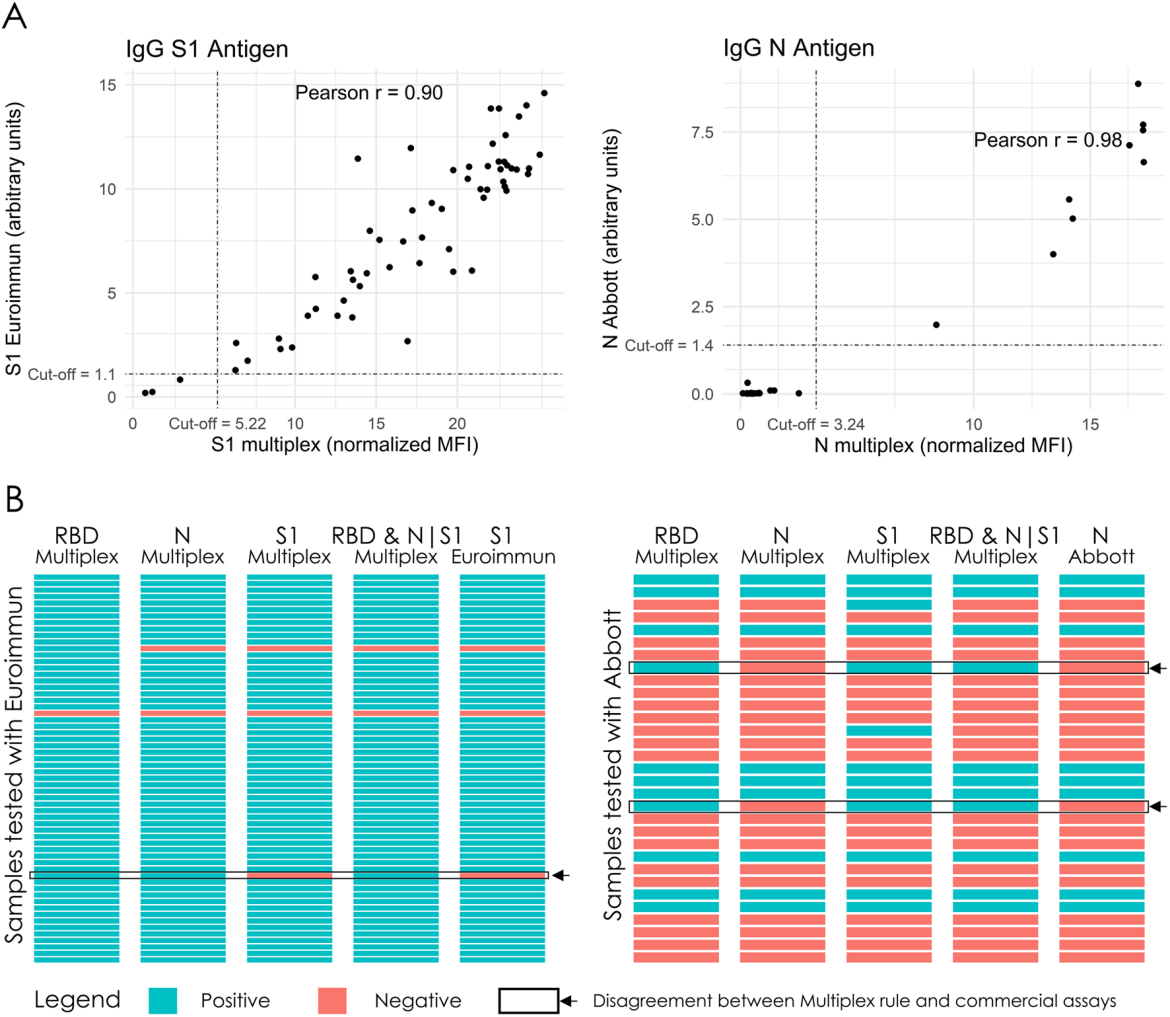
Validation of the multiplex assay against commercial serological assays. (**A**) Scatter plot of the S1 and N single-antigen readouts of the multiplex IgG assay (S1 multiplex and N multiplex) against results from the Euroimmun S1 (n = 60) and Abbott N (n = 31) commercial assays, respectively. The y axis corresponds to commercial assay measurements (arbitrary units), while the x axis to normalized MFI of the multiplex assays. Dotted lines correspond to assay cut-offs for positivity. A Mean + 5 SD cut-off was used for the developed multiplex assays, while the manufacturer’s recommended cut-off was used for the commercial assays. (**B**) Heatmap of diagnostic outcomes depicting the agreement between the multiplex and the commercial assays. The same cut-off values as (**A**) were used for positivity.

The performance of our assay was validated using commercially available antibody tests developed by Abbott and Euroimmun AG against the N and S1 antigens, respectively (Fig. 2). As shown in Fig. 2A, both N and S1 antigen readouts of the multiplex assay were highly correlated with their commercial counterparts (Pearson’s r = 0.98 for N and 0.9 for S1). Their diagnostic agreement (positive/negative call) was 100% when compared to Euroimmun S1 assay and Abbott N assay. A strong agreement was also observed between the commercial assays and the RBD&N|S1 multi-antigen rule (Fig. 2B). Specifically, the RBD&N|S1 rule agreed in 59 out of the 60 samples tested with the Euroimmun S1 assay and in 29 out of the 31 samples tested with the Abbott N assay. Finally, the three samples in which the assays disagreed, were called negative by the commercial assays but showed positive readouts against the remaining two antigens measured in our multiplex assay and were thus correctly called positive by the multi-antigen rule.

### Seroprevalence survey

**Figure 3:**
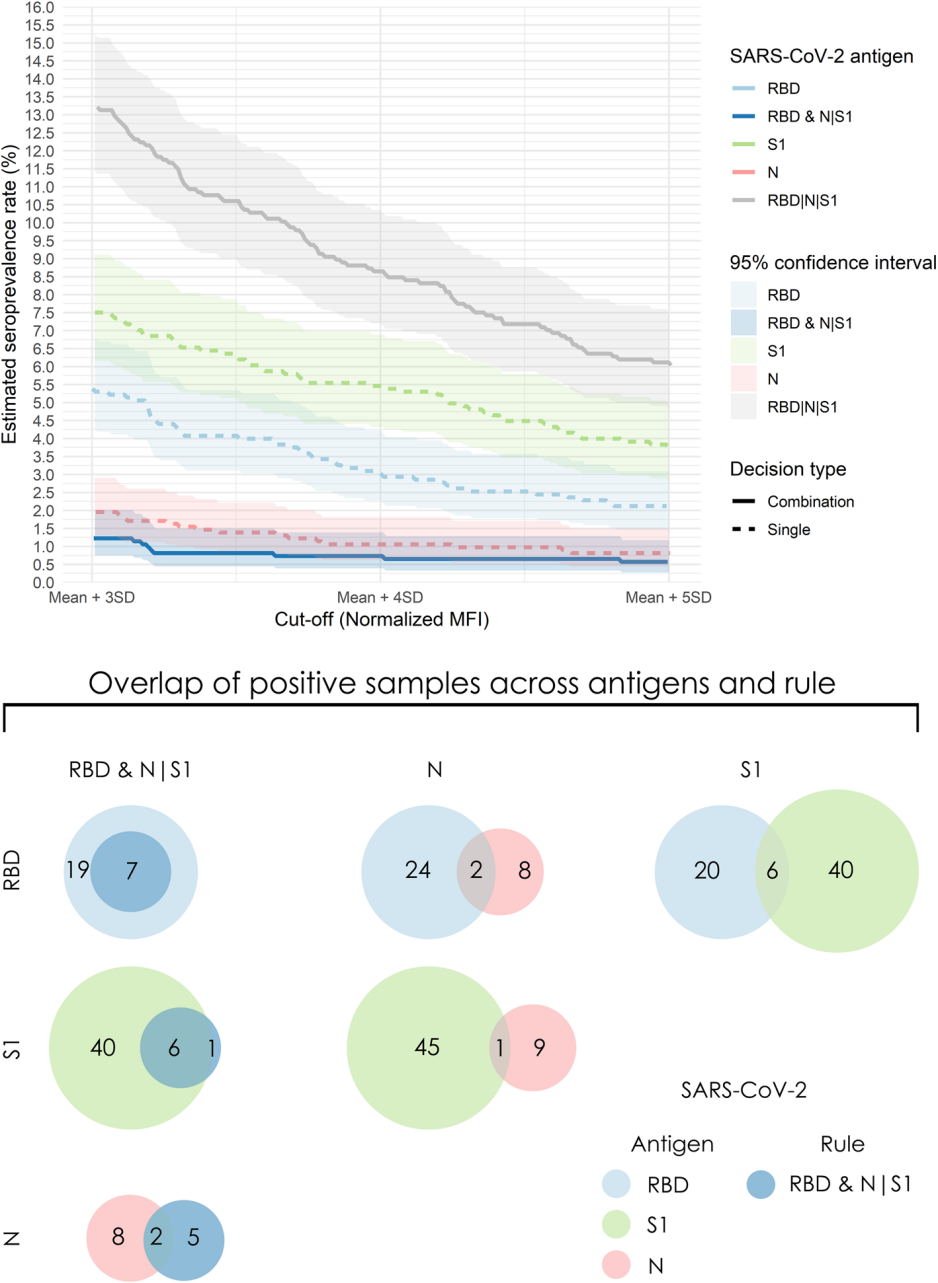
SARS-CoV-2 seroprevalence analysis in 1225 blood donors using the in-house single-antigen readouts or the RBD & N|S1 and RBD|N|S1 multi-antigen rules. (**A**) SARS-CoV-2 estimated seroprevalence rates are presented in the y axis, while different cut-off values are presented in the x axis**.** The cut-off values for each antigen are based on the distribution of normalized MFI of negative samples (**B**) Consensus in detection of SARS-CoV-2 positive individuals using single and multiple-antigen readouts. A stringent cut-off of Mean + 5 SD is used for positivity.

A crucial application of SARS-CoV-2 serological assays is the identification of seroconverted individuals at the population level. Given that exposure to the virus may induce low SARS-CoV-2 related antibodies titers which may also decline over time, a major requirement for this type of analysis is a highly sensitive and specific assay. Therefore, we used the in-house total (IgG/IgA/IgM) SARS-CoV-2 antibody multiplex assay to assess how its high clinical performance translates to estimating seroprevalence in 1225 blood donors with no known history of SARS-CoV-2 exposure. Seroprevalence was calculated based on the single antigen readouts and the RBD & N|S1, RBD|N|S1 multi-antigen rules and was found to be strongly influenced both by the antigen analyzed and the cut-off value used to determine the diagnostic outcome (Fig. 3A). As it can be seen in Fig. 3A, seroprevalence rates from single antigen readouts ranged between 0.8% (N, mean plus 5 SD cut-off, 95% CI 0.4–1.5%) and 7.5% (S1, mean plus 3 SD cut-off, 95% CI 6.0–8.9%), indicating a wide range of potentially indeterminate cases. When using the RBD&N|S1 rule, seroprevalence ranged between 0.6% (mean plus 5 SD cut-off for each antigen, 95% CI 0.3–1.1%) and 1.2% (mean plus 3 SD cut-off, 95% CI 0.7–2.0%), exhibiting a robust profile in line with its robust performance in the clinical setting. Furthermore, we examined the overlap of positive individuals being diagnosed by our single antigens or the RBD&N|S1 rule using the stringent cut-off of mean plus 5 SD (Fig. 3B). We observed a strikingly low agreement between antigens, in that different antigens resulted in vastly different subsets of positive individuals. Specifically, N shared 2 positive samples with RBD (5.9% agreement) and 1 with S1 (1.8% agreement), while S1 and RBD shared 6 positive samples (9.1% agreement). The RBD&N|S1 rule had a total of 7 positive calls, 5 of which were samples with S1 and RBD positive readouts, 1 with RBD and N positive readouts and 1 with all three antigens above the cut-off. The statistical significance of the disagreement between the results of the single antigen readouts and multi-antigen rules was examined using the McNemar’s test and the resulting p-values are presented in Table 5. As it can be seen in Table 5, the striking disagreement between the tests is highly significant, with the only exception being the comparison between the single readout against N and the RBD & N|S1 rule (p-value of 0.4). Finally, we re-analyzed all 1225 samples with an independent commercially available test which detects IgG N-specific antibodies (Abbott). From the 6 samples that were called positive (estimated seroprevalence 0.5%) only 2 also scored positive with the multiplex RBD&N|S1 rule. An analysis of the seroprevalence, agreement rates and overlap of positive samples between the other rules of the multiplex assay (N&RBD|S1, S1&RBD|N) and Abbott’s test results (N-specific IgG) are shown in Supplementary Figs. S5–S7.

**Table 5:** p-values of the McNemar’s test to compare the agreement in positive/negative calls between rules for total antibody detection in the seroprevalence study.

Rule	Rule	p-value
RBD & N|S1	RBD	1.3 × 10^–5^
RBD & N|S1	N	0.4
RBD & N|S1	S1	1.1 × 10^–9^
RBD & N|S1	RBD|N|S1	2.2 × 10^–16^
RBD	N	4.7 × 10^–3^
RBD	S1	9.8 × 10^–3^
RBD	RBD|N|S1	4.3 × 10^–12^
N	S1	9.6 × 10^–7^
N	RBD|N|S1	1.2 × 10^–15^
S1	RBD|N|S1	1.2 × 10^–7^

The cut-off of Mean + 5SD is used for each single and multi-antigen test.

## Discussion

During the COVID-19 pandemic, serological tests play an instrumental role in quantifying seroconversion, seroprevalence, vaccination status and diagnosing recent or prior SARS-COV2 infection. More than 300 assays have been developed using either the N, S1 or RBD antigens and many of them have already reached the market in unforeseen development speeds^4^. However, it is questionable whether single-antigen assays, with strong clinical performance characteristics, show sufficient diagnostic performance in population screening studies^5^. Here, we developed single and multi-antigen assays, evaluated their performance in clinical samples and investigated how the increased performance of multi-antigen assays translates to seroprevalence estimation in a population study.

The developed multiplex platform enabled the simultaneous detection of diverse antibody responses against N, S1 and RBD, within a single diagnostic run. In line with other clinical performance reports, we found a high variability of IgM and IgA responses to different antigens in 77 previously infected SARS-CoV-2 individuals (Supplementary Fig. S3)^14^. In contrast, nearly all positive individuals produced IgG specific antibodies against all three antigens. Other studies have also evaluated N, S1, and RBD antigens in single or multiplex formats and reported similar performance characteristics in samples tested 2 to 3 weeks post infection^6,15–17^. The validation of our single-antigen assays against commercial single-antigen tests (Abbott-N and Euroimmun-S1) showed 100% agreement with our N and S1 readouts (Fig. 2). However, diagnostic outcomes between commercial tests and the RBD&N|S1 rule were slightly different, suggesting that previously infected SARS-CoV-2 individuals may be misdiagnosed by single antigen-based tests (Fig. 2B). In support, the absence of antibodies against certain SARS-CoV-2 antigens is also apparent in studies that have compared assays targeting different antigens^6,17–19^; yet, the biological basis of these observations is currently not well understood.

The rules-based approach combines single antigen readouts, using “AND” and “OR” logic rules, in order to further enhance the specificity, accuracy and robustness of the multi-antigen assay. Such approaches have already been shown to enhance the diagnostic performance of serological assays for common SARS-CoV and for SARS-CoV-2^9^. In our study, we showed that an optimally selected combination of AND plus OR rules can increase specificity and accuracy, with little or no cost on sensitivity, for IgG and total antibody detection in clinical samples (Tables 2, 3). After testing all potential rule combinations the optimal rule was identified as the RBD&N|S1 (Table 2). The optimal rule utilized the best performing readout (RBD antigen) whose specificity was further enhanced by requiring consensus (AND) with either S1, N, or both readouts (Table 3). By using the readout against the RBD antigen as basis, the RBD & N|S1 rule has the added benefit of potentially informing on the presence of neutralizing antibodies as well^11,20^. Additionally, by utilizing the readout against N, the rule can decrease the effects of potential antibody cross-reactivity that exists against the S proteins that are conserved between coronaviruses. Finally, since Spike-based vaccines have shown tremendous efficacy and have received regulatory approval, multiplexing with N has the potential to act as a distinguishing factor between vaccination-induced or natural immunity. Most importantly, the rules-based approach significantly improved assay robustness (i.e. how small deviations of the cut off value affect the diagnostic outcome). The RBD&N|S1 rule resulted in an assay with a statistically significant robust performance profile across a wide range of cut-off values, compared to single-antigen assays, suggesting that this multi-antigen combinatorial assay can achieve optimal performance at lower cut-offs (Fig. 1, Table 4). It is worth noting that while multi-antigen rules exhibited improved performance in IgG and total antibody detection, they did not improve the performance of the IgA and IgM assays (Table 2). However, for the seroprevalence study, we decided to utilize the total (IgG/IgA/IgM) detection assay, in order to correctly identify recently infected individuals who have not yet developed IgG antibodies against the virus.

The use and application of our multiplex strategy in 1255 samples from randomly selected blood donors aims to provide a dataset of experimental settings that mimic routine low-prevalence screening. To the best of our knowledge, this is the first time a multi-antigen serological assay was applied to a seroprevalence study. In contrast to clinical samples, where almost all positive donors showed high antibody titers for all three antigens, a substantial number of donors revealed different responses against the N, S1 and RBD antigens and only one of the 1225 donors showed high antibody titers against all three antigens. With such high dissensus between single-antigen responses, it is obvious that diagnosis of seroconversion in the population becomes challenging (Table 5). Even the highly concordant S1 and RBD antigens, which exhibited an almost perfect agreement in the clinical study, showed only a 9% agreement in the population study (6 out of 66 positive calls) (Fig. 3B). One explanation for this disagreement is that SARS-CoV-2 infected individuals in population-wide screens are mostly asymptomatic, with low antibody titers and unknown time since infection, during which antibodies to specific epitopes may have already waned below the detection limit^22–24^. Another important reason for this large disagreement is the imperfect specificity of single-antigen assays, which can lead to a large number of false predictions in a population screening^5^. For example, assuming 1% seroprevalence and 100% sensitivity, the maximum observed 97.4% single-antigen specificity in the clinical performance study would result in ~ 31 false positive predictions in the serosurvey, whereas just 12 samples are expected to be truly positive^25^. Based on this, we believe that the estimated 7.5% seroprevalence, which was observed in Fig. 3A at mean plus 3 SD with S1, may include mostly false positive predictions. The striking differences in the seroconversion calls between antigens were reflected not only in the wide range of estimated seroprevalence rates (0.6–7.5%) but more importantly in the vastly different subsets of potentially SARS-CoV-2 positive individuals (Fig. 3B). Consistent to our findings, a side-by-side comparison of three fully automated SARS-CoV-2 antibody assays (Abbott against N, Roche against N, and DiaSorin against S1/S2 antigens) showed good agreement in 65 samples from COVID-19 patients, but had profound discrepancies in positive predictions at 1% seroprevalence^26^. Likewise, in an epidemiological study in Iceland, out of the 18,609 individuals tested for total anti-N and anti-S1-RBD antibodies using independent commercial kits, 158 were found positive for either N or S1-RBD but only 39 of them had antibodies against both epitopes^27^. Similar discrepancies between N- and S1-specific serological assays were observed in a large epidemiological study in Spain^28^.

Our results have profound implications regarding seroprevalence rates presented by studies based on single antigen assays, as well as for their diagnostic value. To achieve the most accurate estimate of seroprevalence, an ideal 100% specific assay is required. One approach to increase specificity involves raising the cut-off value of the assay, an approach that IVD manufacturers prefer to adopt to be on the safe side regarding positive predictions. However, raising the cut-off value of single-antigen approaches can lead to a profound underestimation of the seroprevalence rate (as shown in Fig. 3A). A better approach for accurate seroprevalence estimation would be confirmatory testing with an independent assay that uses a different antigenic target^29^. In this paper, we showed that the specificity of the multi-antigen assay can be increased by using a consensus-based strategy, rather than increasing the cut-off value (Table 2). On this front, our rules-based method achieved an almost consistent seroprevalence rate in a wide range of cut-off values between 3 and 5 standard deviations above the mean (Fig. 3A, Table 4). Consequently, we believe that the RBD&N|S1 rule combined with a mean plus 5 SD threshold provides a realistic estimation of seroprevalence in the community and accurate identification of seroconverted individuals. Notably, in our study we used cut-off thresholds between 3 and 5 SDs whereas the manufacturer’s recommended 1.4 cut-off value for the IgG N-specific Abbott test was calculated to correspond to more than 10 SDs above the negative mean. Such high cut-off values can undermine sensitivity and result in the underestimation of the true seroprevalence rate. When using the rules-based multiplex assay (RBD&N|S1) as reference the positive predictive value of the IgG N-specific Abbot test was only 29% (2 out of 7 detected), despite both assays showing excellent agreement in clinical samples (Supplementary Fig. S7). An important limitation of such comparisons between serological assays in population-wide surveys is the fact that there is no gold standard method to identify individuals exposed to SARS-CoV-2.

Whilst offering improvements in diagnostic performance and population-wide studies, multi-antigen based serological assays do come with some limitations. Assuming that antibodies to different antigens have different decay rates, the consensus-based rules have the potential caveat of misdiagnosing positive individuals, whose antibody responses to two out of three antigens have waned below detection levels and subsequently underestimate the seroprevalence rate in the population. Additionally, population’s exposure to the virus, if based solely on antibody tests (both multi- and single-antigen), can be underestimated since asymptomatic infection could cause T cell immunity without detectable seroconversion. Finally, multiplex technologies are not as accessible as their more traditional single ELISA counterparts which makes them a less popular choice especially among countries with preexisting economic challenges during a rapid onset, global pandemic. The reduced usage of resources and time achieved by multiplexing may counterbalance some of these issues in the near future.

In conclusion, our study has demonstrated that serological assays based on single antigens, while good at diagnosing infected individuals in a clinical setting, may not be ideal in low seroprevalence, population-wide COVID19 screens, where low antibody responses are expected. A multi-antigen consensus-based approach for diagnostic decisions can provide a better alternative in this context, and benefit already existing multi-antigen assays through its enhanced specificity and reduced dependency on cut-off values. We believe that such multi-antigen approaches should be performed in a single multiplex assay, thus diminishing possible differences attributed to operational issues of independent assay formats^14^. The embrace of multiplex assays or multiple single antigen-based assays, by the scientific community, for epidemiological studies can eventually lead to more accurate and reliable results regarding SARS-CoV-2 spread in the population.

## Methods

### Serum samples

A total of 155 clinical serum samples were analyzed, of which 78 were negative as were banked sera collected prior to the COVID-19 pandemic (2018 up until November 2019) and 77 were positive samples from PCR-confirmed SARS-CoV-2 individuals collected at least 2 weeks after SARS-CoV-2 infection. Nineteen additional serum samples from PCR-confirmed SARS-CoV-2 individuals were used for assay validation against other commercial SARS-CoV-2 serological assays. For the general population screening, we obtained 1225 serum samples between the 23rd and 25th week of 2020, e.g. 5 weeks after lockdown in Greece that took place between the 13th and 18th week of 2020; blood donors were from 13 different geographical regions in Greece (26% female; median age, 42 years; range 17–65). Eligibility for donation included an extended detailed questionnaire for previous possible signs of infection during 2020. All samples were acquired under approved clinical protocols and informed consent (see Ethics statement). The detailed list of serum samples used throughout the study is presented in Table 1.

### Multiplex immunoassay development

A magnetic bead-based immunoassay was developed using the xMAP Luminex technology against SARS-CoV-2 antigens N, S1 and RBD. One antigen from each one of the four endemic coronaviruses was also included in the assay. Specifically, the S1 subunit of HCoV-HKU1, HCoV-229E and HCoV-NL63 and the S1 + S2 subunits from HCoV-OC43 were used. The SARS-CoV-2N and S1 antigens were purchased from the Native Antigen Company (Kidlington, UK). All other antigens were from Sino Biological Europe GmbH (Eschborn, Germany). Each antigen was covalently coupled to a distinct magnetic bead region (Luminex Corp, Austin, Texas) by carbodiimide coupling at a ratio of 15 μg per 5 million beads^21^. Coupling efficiency was confirmed by incubation of 5000 beads from each coupled region with a phycoerythrin-conjugated anti-6× HisTag antibody (Abcam, Cambridge, UK) at a concentration of 32 μg/mL for 15 min at room temperature. Coupled beads were mixed to a final concentration of 50 beads/μL and stored in PBS supplemented with 1% bovine serum albumin, 0.02% Tween-20 and 0.05% sodium azide at 4 °C until use. For analysis of serum samples, 25 μL of the bead mix (corresponding to 1250 beads per antigen) were added to each well of a 96-well plate, washed twice with 100 μL Assay Buffer (PBS supplemented with 1% BSA and 0.05% sodium azide) and incubated with 50 μL of serum diluted in LowCross-Buffer^®^ (CANDOR Bioscience GmbH, Wangen, Germany) for 2 h at room temperature in a plate shaker (900 rpm). A serum dilution of 1/400 was used for testing all immunoglobulin types except for IgA that was assayed at a 1:100 serum dilution was used. Unbound material was removed by two washes with 100 μL assay buffer and beads were incubated with 20 μL of biotinylated anti-human immunoglobulin antibodies (Jackson ImmunoResearch Europe Ltd, Ely, UK) for 1 h at room temperature in a plate shaker (900 rpm). Antibodies were diluted in assay buffer at 1:1600 for IgG/IgM/IgA, 1:800 for IgG, 1:3200 for IgA and 1:800 for IgM. Beads were washed twice with 100 μL assay buffer and incubated with streptavidin R-phycoerythrin (Jackson ImmunoResearch Europe Ltd, Ely, UK) diluted 1:100 in assay buffer for 15 min at room temperature in a plate shaker (900 rpm). Beads were washed again as before, reconstituted in 130 μL assay buffer, and measured in a FLEXMAP 3D instrument (Luminex Corp, Austin, Texas). Instrument settings included standard PMT, 100 μL sample volume, a bead count of 50 beads per antigen and doublet discrimination gate set at 3000–20,000.

### Diagnostic assay performance analysis

The Median Fluorescent Intensity (MFI) values of each SARS-CoV-2 antigen were first divided by the average MFI of the negative control samples (made from a pool of negative sera) for the same antigen. These “normalized MFI” values were used in all subsequent calculations. Cut-off values for determining the diagnostic outcome (positive/negative) regarding the presence (or not) of SARS-CoV-2 specific antibodies were calculated for each SARS-CoV-2 antigen based on its distribution at the negative samples. The diagnostic performance of the assay was assessed for cut-off values ranging from mean *plus* one standard deviation (SD) up to mean plus five SD. Performance was evaluated in terms of sensitivity $\left(\frac{true \,positives}{positives}\right)$, specificity $\left(\frac{true\, negatives}{negatives}\right)$ and accuracy, while the corresponding 95% confidence intervals (CI) were calculated using the Wilson approximation^30^. Furthermore, for each antibody isotype and antigen, the Receiver Operating Characteristic (ROC) curves and the corresponding area under the curve (AUC) was calculated (see Supplementary Fig. S4).

### Rules-based method

A rules-based method was developed to combine the readouts of the multi-antigen assay. First, the single antigen “normalized MFI” values were transformed to positive/negative predictions by comparing them with the appropriate cut-off value. Then, logic circuits that utilize the “AND” (represented by the symbol “&”) and “OR” (represented by the symbol “|”) logic gates were implemented. These logic circuits take as input the single-antigen predictions and output the final rules-based prediction that corresponds to a positive/negative call for the presence of SARS-CoV-2 specific antibodies. All possible simple circuits that could be formed using the “AND” and “OR” logic gates to combine the predictions of the RBD, S1 and N predictions were examined.

### Statistical analysis

In order to calculate the power of the proportion test that compares the specificity and sensitivity between single-antigen tests and rules the R package pwr was used. The null hypothesis was that the proportions are equal, while the alternative was that they are not. The sample sizes were 78 negative samples for the comparison of specificity and 77 positive samples for the comparison of sensitivity. The significance level was set at 0.05. For the significance analysis of the robustness, we used the McNemar’s test provided in the R package DTComPair, to determine if a diagnostic test produces significantly different results when different cut-off values are utilized. The significance threshold was set at 0.05. In order to evaluate the robustness of sensitivity and specificity the contingency tables across different cut-offs were used for the positive and negative samples, respectively. Finally the p-values of the test were calculated from the chi-squared distribution. In the seroprevalence study, the McNemar’s test was used to determine if there is a statistically significant difference in the results of different diagnostic assays during an epidemiological study. Overall, p-values < 0.05 were considered significant^31,32^.

### Assay validation

Assay validation was performed in two separate subsets of matched clinical samples against two widely used, commercially available SARS-CoV-2 antibody tests developed by Euroimmun (Euroimmun Medizinische Labordiagnostika AG, Lubeck, Germany) and Abbott (Abbott Diagnostics, Illinois, USA), which detect IgG antibodies against S1 and N respectively (Supplementary Table S8). Single antigen readouts for S1 and N from our IgG multiplex assay were plotted against the results of the commercial tests and the Pearson’s correlation coefficient was calculated. For determining the diagnostic outcome (positive/negative calls) we used the mean plus five SD cut-off values for our assays and the manufacturer’s recommended cut-offs for the commercial assays (1.1 for Euroimmun and 1.4 for Abbott).

### Population-level analysis

For the analysis of the 1225 samples from blood donors, the total (IgG/IgA/IgM) assay was used, and the diagnostic outcome was assessed across cut-off values ranging from mean plus 3 SD to mean plus 5 SD from the negative sample distribution. To identify seroconverted individuals, the diagnostic outcomes using the mean plus 5 SD cut off values were utilized. Frozen back-up samples (n = 1225) were sent to the Immunology Laboratory of the National Public Health Organization, Athens, Greece and analyzed for the presence of IgG antibodies against the N antigen using the Abbott IgG assay with the ARCHITECT i2000SR analyzer (Abbott, Illinois, United States). The manufacturer’s recommended cut-off (1.4) was used to determine positivity.

### Ethics statement

Sampling from SARS-CoV-2 positive individuals was done with informed consent and under approved institutional and ethics board-approved clinical protocols, conducted in full compliance with the principles of Good Clinical Practice and the Declaration of Helsinki (University Hospital of Patras EC 164/27.04.2020 and IRB 216/08.05.2020, Alexandra General Hospital NCT04408209 trial). Negative samples were leftover material acquired before the COVID-19 pandemic (2019, 2018), which were stored and used for scientific purposes after local ethical approval and informed consent (EC 344/18.9.06). The population-level study was approved by the Research Ethics Committee of the University of Patras (Ref. Number 6099) and all participants gave written informed consent.

## Supplementary Information

Supplementary Information.

## Data Availability

All data and code required to reproduce the results presented in the manuscript are available at https://github.com/BioSysLab/COVID-19-Multiplex-Assay.

## References

[1] Lipsitch M, Swerdlow DL, Finelli L (2020). Defining the epidemiology of covid-19—Studies needed. N. Engl. J. Med..

[2] Weinstein MC, Freedberg KA, Hyle EP, Paltiel AD (2020). Waiting for certainty on covid-19 antibody tests—At what cost?. N. Engl. J. Med..

[3] Greenland S (1996). Basic methods for sensitivity analysis of biases. Int. J. Epidemiol..

[4] Deeks JJ (2020). Antibody tests for identification of current and past infection with SARS-CoV-2. Cochrane Database Syst. Rev..

[5] Bryant JE (2020). Serology for SARS-CoV-2: Apprehensions, opportunities, and the path forward. Sci. Immunol..

[6] Randad PR (2020). COVID-19 serology at population scale: SARS-CoV-2-specific antibody responses in saliva. MedRxiv..

[7] Rosado J (2020). Serological signatures of SARS-CoV-2 infection: Implications for antibody-based diagnostics. MedRxiv.

[8] den Hartog G (2020). SARS-CoV-2-specific antibody detection for sero-epidemiology: A multiplex analysis approach accounting for accurate seroprevalence. J. Infect. Dis..

[9] Dobaño C (2020). Highly sensitive and specific multiplex antibody assays to quantify immunoglobulins M, A and G against SARS-CoV-2 antigens. BioRxiv..

[10] Ayouba A (2020). Multiplex detection and dynamics of IgG antibodies to SARS-CoV2 and the highly pathogenic human coronaviruses SARS-CoV and MERS-CoV. J. Clin. Virol..

[11] Mariën J (2021). Evaluating SARS-CoV-2 spike and nucleocapsid proteins as targets for antibody detection in severe and mild COVID-19 cases using a Luminex bead-based assay. J. Virol. Methods.

[12] de Assis RR (2020). Analysis of SARS-CoV-2 antibodies in COVID-19 convalescent blood using a coronavirus antigen microarray. BioRxiv..

[13] Lv H (2020). Cross-reactive antibody response between SARS-CoV-2 and SARS-CoV infections. Cell Rep..

[14] Whitman JD (2020). Evaluation of SARS-CoV-2 serology assays reveals a range of test performance. Nat. Biotechnol..

[15] Liu W (2020). Evaluation of nucleocapsid and spike protein-based enzyme-linked immunosorbent assays for detecting antibodies against SARS-CoV-2. J. Clin. Microbiol..

[16] Lassaunière R (2020). Evaluation of nine commercial SARS-CoV-2 immunoassays. MedRxiv..

[17] Okba NMA (2020). Severe acute respiratory syndrome coronavirus 2-specific antibody responses in coronavirus disease patients. Emerg. Infect. Dis..

[18] Zhang G, Nie S, Zhang Z, Zhang Z (2020). Longitudinal change of severe acute respiratory syndrome coronavirus 2 antibodies in patients with coronavirus disease 2019. J. Infect. Dis..

[19] Grzelak L (2020). A comparison of four serological assays for detecting anti–SARS-CoV-2 antibodies in human serum samples from different populations. Sci. Transl. Med..

[20] Terpos E (2020). Anti–SARS-CoV-2 antibody responses in convalescent plasma donors are increased in hospitalized patients; subanalyses of a phase 2 clinical study. Microorganisms.

[21] Trivedi SU (2019). Development and Evaluation of a multiplexed immunoassay for Simultaneous detection of serum IgG antibodies to six human coronaviruses. Sci. Rep..

[22] Amanat F (2020). A serological assay to detect SARS-CoV-2 seroconversion in humans. Nat. Med..

[23] Long QX (2020). Clinical and immunological assessment of asymptomatic SARS-CoV-2 infections. Nat. Med..

[24] Ibarrondo FJ (2020). Rapid decay of anti-SARS-CoV-2 antibodies in persons with mild covid-19. N. Engl. J. Med..

[25] Diamandis P, Prassas I, Diamandis EP (2020). Antibody tests for COVID-19: Drawing attention to the importance of analytical specificity. Clin. Chem. Lab. Med..

[26] Perkmann T (2020). Side by side comparison of three fully automated SARS-CoV-2 antibody assays with a focus on specificity. MedRxiv.

[27] Gudbjartsson DF (2020). Humoral immune response to SARS-CoV-2 in Iceland. N. Engl. J. Med..

[28] Pollán M (2020). Prevalence of SARS-CoV-2 in Spain (ENE-COVID): A nationwide, population-based seroepidemiological study. Lancet.

[29] NIH.GOV (2019). Treatment Guidelines Panel. Coronavirus Disease 2019 (COVID-19) Treatment Guidelines.

[30] Wilson EB (1927). Probable Inference, the law of succession, and statistical inference. J. Am. Stat. Assoc..

[31] Kim S, Lee W (2017). Does McNemar’s test compare the sensitivities and specificities of two diagnostic tests?. Stat. Methods Med. Res..

[32] Fagerland MW, Lydersen S, Laake P (2013). TheMcNemar test for binary matched-pairs data: Mid- p and asymptotic are better than exact conditional. BMC Med. Res. Methodol..

