# Supplementary material for: Accurate SARS-CoV-2 seroprevalence surveys require robust multi-antigen assays
Source: Sci Rep. 2021 Mar 23;11:6614. doi: 10.1038/s41598-021-86035-2 (PMC7988055; doi:10.1038/s41598-021-86035-2)
Supplement: Supplementary file 1 — Supplementary Information. [file 41598_2021_86035_MOESM1_ESM.pdf]

Title: Accurate SARS-CoV-2 seroprevalence surveys require robust multi-antigen assays

Authors: Christos Fotis[1+], Nikolaos Meimetis[1+], Nikos Tsolakos[2+], Marianna Politou[3+], Karolina Akinosoglou[4+], Vaia Pliaka[2], Angeliki Minia[2], Evangelos Terpos[3], Ioannis P. Trougakos[5], Andreas Mentis[6], Markos Marangos[4], George Panayiotakopoulos[7,8], Meletios A. Dimopoulos[3], Charalampos Gogos[4], Alexandros Spyridonidis[9#*], Leonidas G. Alexopoulos[1,2#*]

1. Biomedical Systems Laboratory, National Technical University of Athens, Athens, Greece
2. ProtATonce Ltd, Demokritos Science Park, Athens, Greece
3. Department of Clinical Therapeutics, Alexandra General Hospital, National and Kapodistrian University of Athens, Athens, Greece
4. Department of Internal Medicine, Division of Infectious Diseases, University Hospital of Patras, Patras, Greece
5. Department of Cell Biology and Biophysics, Faculty of Biology, National and Kapodistrian University of Athens, Athens, Greece
6. Medicinal Microbiology Laboratory, Hellenic Pasteur Institute, Athens, Greece
7. Pharmacology Laboratory, University of Patras, Patras, Greece
8. National Public Health Organization, Athens, Greece
9. Department of Internal Medicine, BMT Unit and CBMDP Donor Center, University of Patras, Patras, Greece

*These co-first authors contributed equally to this article

[#]These authors were co–principal investigators

[*] Corresponding authors: E-mails: leo@mail.ntua.gr (LGA) and spyridonidis@upatras.gr (AS)

Supplementary results

S1.1 Optimization of assay parameters

Serum dilution and antibody detection concentration were optimized during assay development using a small subset of well-defined positive samples for prior SARS-CoV-2 infection and negative serum samples (Supplementary Figures S1 & S2). Samples were assessed at four two-fold serial dilutions (1:100, 1:200, 1:400 and 1:800). A serum dilution of 1:400 was found to provide the best ratio of positive to negative MFI values in total (IgG/IgM/IgA), IgG and IgM isotype detection and was chosen for subsequent experiments. For IgA detection, the optimal serum dilution was 1:100. The biotinylated detection antibodies were also tested at two-fold serial dilutions from 1:800 to 1:6,400 and the signal-to-noise ratio (SnR) of each serum sample to a blank sample was calculated. The antibody concentrations that increased SnR values in positive samples and/or decreased SnR values in negative samples were

considered optimal. A dilution of 1:800 was chosen for IgG and IgM isotypes, while antibodies against total and IgA isotypes were used at 1:1,600 and 1:3,200 dilution, respectively.

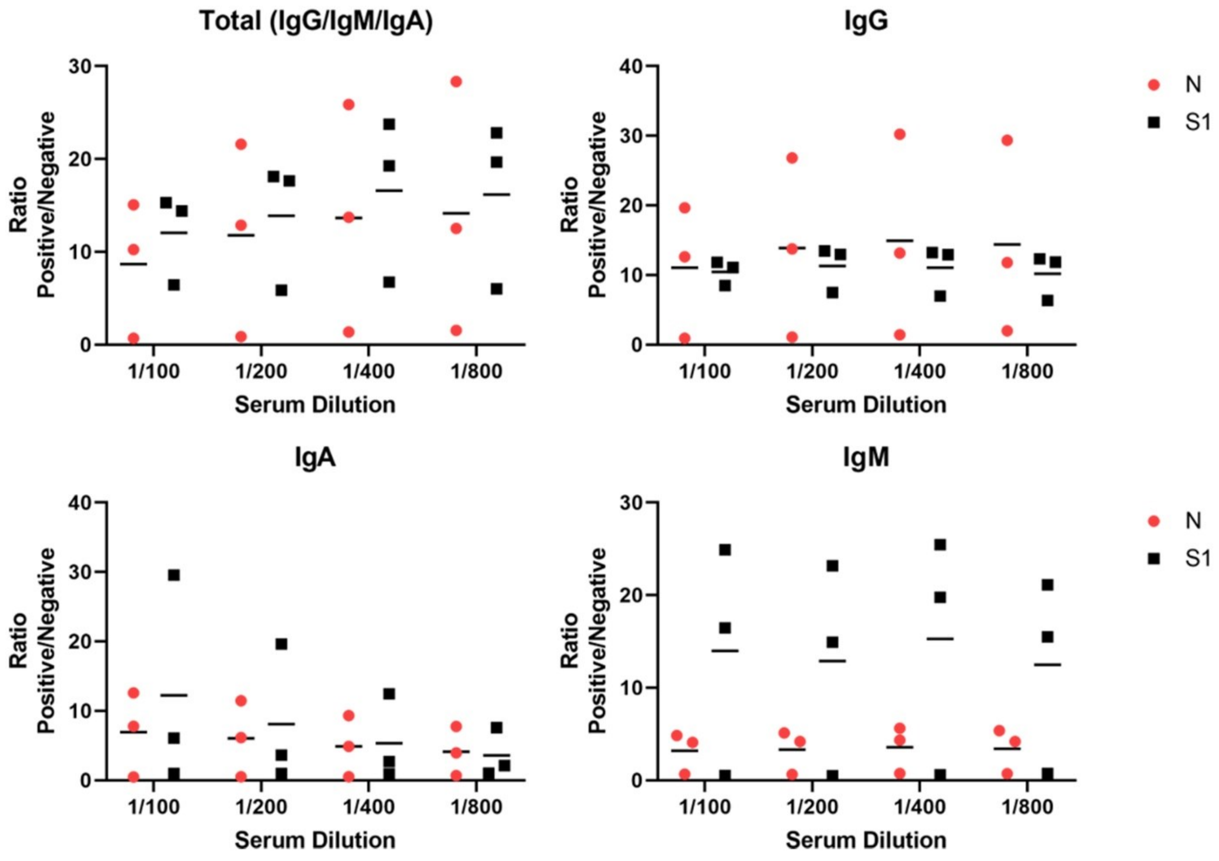

Supplementary Figure S1. Serum Dilution Optimization. Serum from three SARS-CoV-2 positive samples and four negative samples cases were tested at four dilutions and the ratio of the MFI in each positive case to the average MFI from the negative cases was calculated and plotted for N and S1 antigens. Black lines represent the mean of the three positive samples.

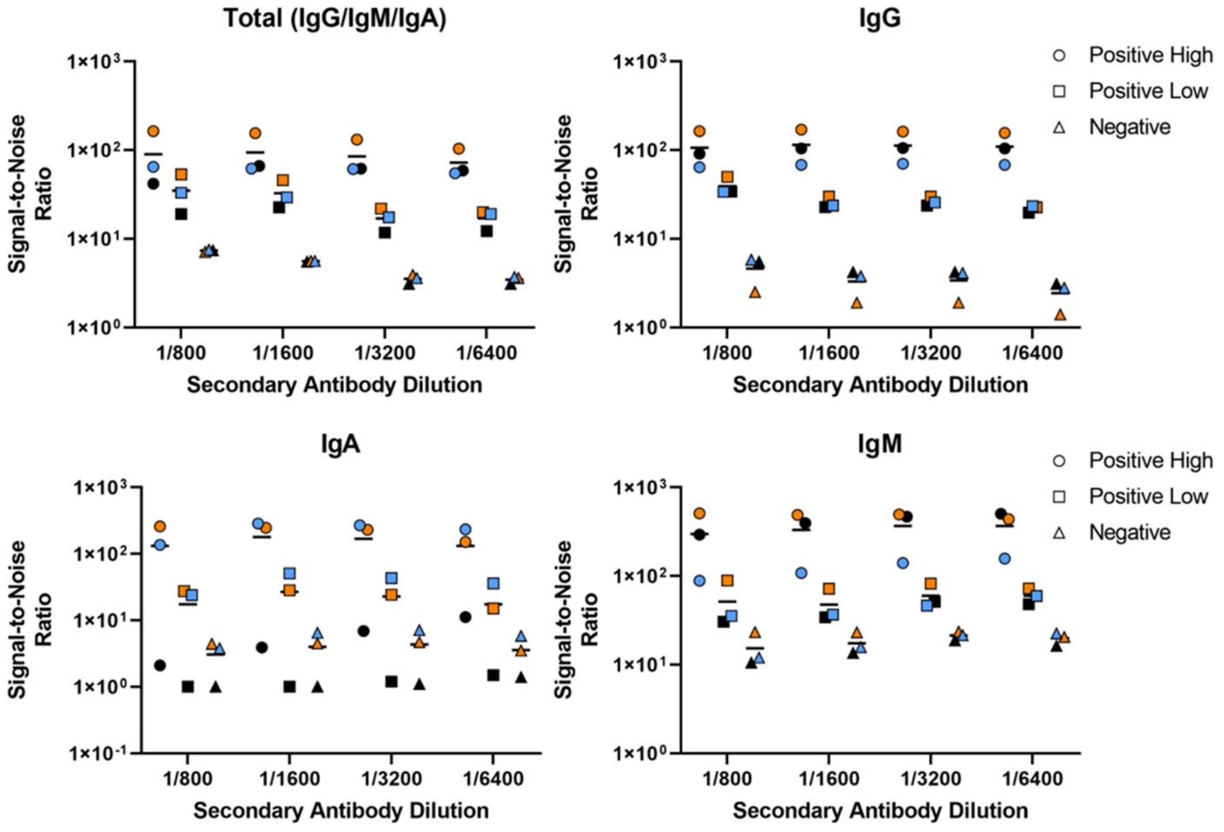

Supplementary Figure S2. Secondary Antibody Dilution Optimization. A SARS-CoV-2 positive serum with high antibody levels (positive high - circles), a SARS-CoV-2 positive serum with low antibody levels (positive low - squares), a negative serum (triangles) and a blank sample (serum diluent only) were tested at four two-fold serial dilutions of the secondary antibody. For each positive and negative sample, the signal-to-noise ratio against the blank sample was calculated for N (black), S1 (orange) and RBD (blue) and plotted against the antibody dilutions. Black lines represent the mean of the three antigens in each condition.

S1.2 Analytical assay performance

For assessment of analytical assay performance (intra- and inter-assay variability) as well as for quality control purposes, we generated two control samples, a positive control made from a pool of sera from PCR-confirmed COVID-19 cases and a negative control made from a pool of negative sera. These samples were tested in quadruplicates in the same and in separate 96-well plates at different days and MFI values were used to calculate the coefficient of variation (CV%) for intra- and inter-assay variability. Also, the ratio of the average MFI values of the positive against the negative control for the SARS-CoV-2 N, S1 and RBD antigens was calculated and used as an indicator for the proper performance of the assay procedure by the end-user. Assay performance was considered successful when a ratio of three or greater for all three antigens was achieved. CV% values for the three SARS-CoV-2 antigens were below 20% across all isotypes (Supplementary Table S1). The IgG assay performed better across all antigens, while the highest variability was observed in the antigens from the endemic coronaviruses in the total IgG/IgM/IgA assay.

Supplementary Table S1. Intra- and Inter-assay variability, measured as % coefficient of variation.

Antibody Isotype	Variability	Sample	SARS-CoV-2 N	SARS-CoV-2 S1	SARS-CoV-2 RBD	HKU1 S1	NL63 S1	229E S1	OC43 S1+S2
	Intra-assay	Positive serum	8.2	8.2	8.2	20.3	23.5	20.7	12.2

Total (IgG/IgM/IgA)		Negative serum	15.2	13.8	8.3	18.0	19.9	16.2	13.7
	Inter-assay	Positive serum	4.3	4.3	4.1	11.5	13.7	11.0	5.6
		Negative serum	15.8	16.5	8.2	28.9	30.6	24.4	21.3
IgG	Intra-assay	Positive serum	0.9	0.7	1.1	2.5	3.6	2.5	1.0
		Negative serum	4.1	3.2	5.9	4.0	5.1	3.2	3.1
	Inter-assay	Positive serum	1.0	1.4	1.3	3.2	3.6	2.5	1.2
		Negative serum	1.6	2.5	4.0	3.2	3.7	2.5	2.7
IgA	Intra-assay	Positive serum	7.4	8.2	8.6	12.8	13.7	12.3	9.8
		Negative serum	4.6	6.7	4.7	5.5	6.2	5.1	4.1
	Inter-assay	Positive serum	10.0	11.3	11.3	17.5	17.7	16.1	13.2
		Negative serum	4.9	7.2	5.0	7.1	8.0	6.5	4.6
IgM	Intra-assay	Positive serum	2.4	1.9	2.3	7.0	5.0	4.9	4.0
		Negative serum	3.8	4.0	3.4	4.1	8.7	5.2	3.7
	Inter-assay	Positive serum	2.8	2.3	1.8	3.9	2.2	4.5	4.0
		Negative serum	3.9	8.5	5.6	7.1	6.2	7.7	6.9

S1.3 Antibody responses to SARS-CoV-2 and endemic human coronaviruses in clinical samples

A total of 155 serum samples from 77 PCR-confirmed COVID-19 cases and 78 pre-epidemic individuals were screened for the existence of reactive antibodies against antigens of the five different coronaviruses (Supplementary Table S2). SARS-CoV-2 infection was deemed asymptomatic and mild needing no hospitalization in 8% and 60% of the cases, respectively, whereas 32% of the participants required hospitalization. Antibody detection was performed at a median of 46 days (range 13-87) post SARS-CoV-2 infection. Supplementary Figure S3 shows the reactivity of sera to each antigen for the different antibody isotypes. Antibody levels against the SARS-CoV-2 antigens N, S1 and RBD were significantly higher in the SARS-CoV-2 infected population as compared to samples from non-infected individuals ($p<0.001$, Supplementary Table S4); this finding was true for all isotypes. The analyzed blood samples from SARS-CoV-2 tested positive donors also showed significantly high antibody titers against the HCoV-OC43 S1/S2 antigens in all isotypes and against the HCoV-HKU1 S1 antigen in IgG detection, possibly as a result of antibodies cross-reactivity against S proteins that are conserved between SARS-CoV-2, HCoV-HKU1 and HCoV-OC43 coronaviruses.

Additionally, a power calculation was conducted to investigate the ability of the assay to discriminate between sero-positives and sero-negatives, using a t-test with a significance threshold of 0.001. The effect size based on the difference between the means of the negative and positive distributions, was calculated using Cohen's d ($=\frac{\overline{x_1}-\overline{x_2}}{s}$), in which the difference in means is divided by the pooled standard deviation of the two independent samples. As it is expected by observing Supplementary figure S3, there is a large effect size, resulting in a 100% power. This means that the single antigen readouts of the multiplex assay can easily distinguish sero-positive from sero-negative cases given the sample size (Supplementary Table S3).

We further assessed the correlation of the normalized MFI values between antigens in both positive and negative cases (Supplementary Table 5). The SARS-CoV-2 S1 and RBD antigens-related data correlated strongly across all Ig isotypes in COVID-19 positive samples with Pearson's correlation coefficient (r) values > 0.96; a high correlation

was also observed between N and S1 or RBD readouts in IgG and total antibody assays (r values between 0.72-0.82) in COVID-19 positive samples. Readouts between the N and S1 or RBD antigens correlated poorly in IgA and IgM detection. Results from negative samples showed no correlation across all antigen comparisons and Ig isotypes with the notable exception of the S1 and RBD antigens in IgM isotype (r=0.78).

Supplementary Table S2. List of samples used throughout this study.

Cohort type	Sample type	Sample source	No of samples	Demographics		
				Median age (range)	Gender	Days post infection (range)
Clinical	Positive serum (SARS-CoV-2 PCR-confirmed cases)	Alexandra General Hospital, Athens	60	49 (20-74)	65% M 35% F	46 (13-87)
		University Hospital of Patras	17			
	Negative serum (banked samples from 2018-2019)	University Hospital of Patras	78	57 (17-71)	51% M 49% F	N/A
Population screen	Serum from blood donors	Multi-center/ University of Patras	1225	42 (17-65)	74% M 26% F	N/A

Supplementary Table S3. Power calculation regarding the ability of the assay to discriminate between the antibody levels of sero-positives and sero-negatives, using the N, S1 and RBD single antigen readouts. The number of positives is n = 77 and the number of negatives n = 78.

Antigen	Power (%)	Effect Size
N	100	3.725
S1	100	3.644
RBD	100	3.773

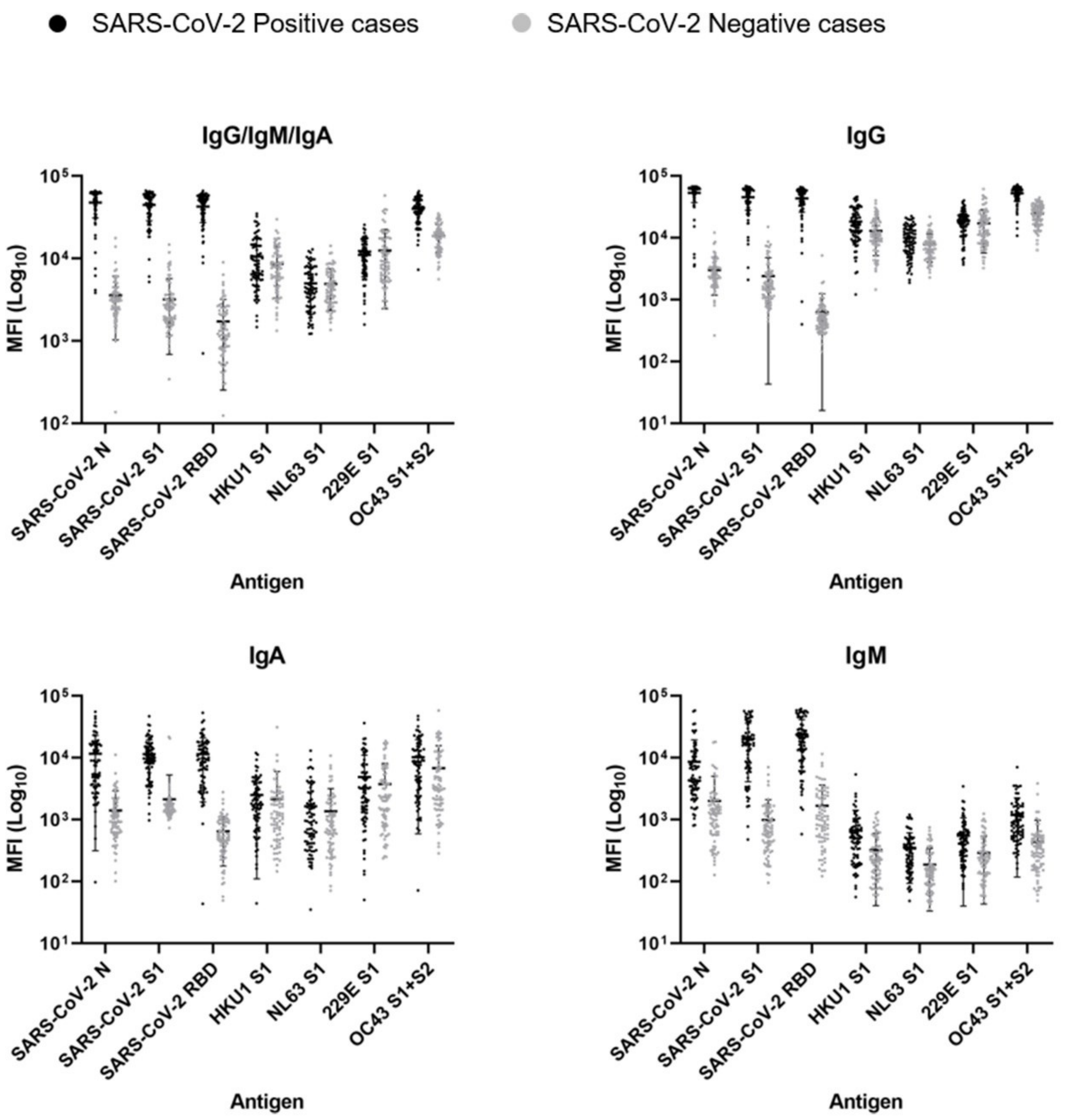

Supplementary Figure S3. Antibody responses against SARS-CoV-2 N, S1 and RBD antigens and 4 endemic coronaviruses antigens in serum samples from SARS-CoV-2 PCR-tested positive and negative (banked blood samples from 2018-2019) individuals. The Median Fluorescence Intensities (MFI, log10 scale) measured in positive and negative samples were plotted for each antigen. Graphs indicated the different antibody isotypes tested. Mean plus standard deviation (SD) error bars are presented.

Supplementary Table S4. Antibody responses to coronavirus antigens in SARS-CoV-2 positive and negative cases.

		SARS-CoV-2 Positive Cases		SARS-CoV-2 Negative Cases		
**Isotype**	**Antigen**	**Mean**	**SD**	**Mean**	**SD**	**P-Value**
Total (IgG/IgM/IgA)	SARS-CoV-2 N	47598.8	16571.5	3571.9	2541.0	<0.000001
	SARS-CoV-2 S1	44681.6	15960.5	3188.2	2504.0	<0.000001
	SARS-CoV-2 RBD	42692.9	15336.8	1721.6	1467.9	<0.000001
	HKU1 S1	10564.6	7393.9	8587.7	5259.1	0.185844

	NL63 S1	5011.9	2905.8	4918.9	2566.9	0.950362
	229E S1	11056.3	5040.5	12465.3	10001.0	0.345655
	OC43 S1+S2	40071.6	12817.1	18794.9	6972.1	<0.000001
	SARS-CoV-2 N	53384.7	16219.0	3017.1	1824.5	<0.000001
	SARS-CoV-2 S1	45661.3	17638.3	2405.4	2362.3	<0.000001
	SARS-CoV-2 RBD	43344.2	17129.8	628.3	612.2	<0.000001
IgG	HKU1 S1	18405.4	10656.9	12968.2	7818.9	0.001355
	NL63 S1	10008.0	5235.7	7828.3	3758.6	0.198085
	229E S1	18640.9	8172.8	17179.1	11478.5	0.387976
	OC43 S1+S2	52576.7	13693.9	24740.7	9010.3	<0.000001
	SARS-CoV-2 N	11626.9	11311.3	1405.7	1518.3	<0.000001
	SARS-CoV-2 S1	11443.1	8100.1	2126.1	3151.4	<0.000001
	SARS-CoV-2 RBD	11455.2	9523.9	646.2	469.3	<0.000001
IgA	HKU1 S1	2497.7	2387.3	2143.3	3836.7	0.72526
	NL63 S1	1644.2	2243.8	1367.7	1795.8	0.783876
	229E S1	4866.8	6111.9	3758.0	4360.1	0.271624
	OC43 S1+S2	10162.1	9569.3	6781.4	8937.7	0.000826
	SARS-CoV-2 N	8637.6	10910.7	1997.5	3019.2	<0.000001
	SARS-CoV-2 S1	20217.3	16141.8	986.4	1112.8	<0.000001
	SARS-CoV-2 RBD	23718.1	17758.2	1679.1	1928.3	<0.000001
IgM	HKU1 S1	675.0	741.6	321.3	280.7	0.756783
	NL63 S1	351.6	258.9	187.2	154.0	0.885512
	229E S1	552.3	512.4	290.5	247.2	0.818666
	OC43 S1+S2	1187.1	1068.8	439.5	534.0	0.512724

Supplementary Table S5. Correlation of normalized MFI values between SARS-CoV-2 antigens for the different antibody isotypes in positive and negative cases.

**Isotype**	**Comparison**	**SARS-CoV-2 Positive Cases**	**SARS-CoV-2 Negative Cases**
	N *vs* S1	0.82	0.29
Total (IgG/IgM/IgA)	N *vs* RBD	0.81	0.32
	S1 *vs* RBD	0.98	0.23
IgG	N *vs* S1	0.75	0.22

	N *vs* RBD	0.72	0.38
	S1 *vs* RBD	0.98	0.34
	N *vs* S1	0.27	0.56
IgA	N *vs* RBD	0.24	0.21
	S1 *vs* RBD	0.96	-0.03
	N *vs* S1	0.39	0.37
IgM	N *vs* RBD	0.35	0.27
	S1 *vs* RBD	0.96	0.78

S1.4 Correlation of SARS-CoV-2 antibody responses to antibody levels from other infectious agents

For assessing interfering factors, we used results from 66 pre-epidemic fully characterized sera for the presence of antibodies against Cytomegalovirus (CMV), Epstein-Barr virus (EBV), Hepatitis A (HAV) and B (HBs) and Toxoplasma (Toxo) (Supplementary Table S6). CMV IgG levels positively correlated with SARS-CoV-2 S1 IgG and total (IgG/IgM/IgA) antibodies ($p<0.01$) but showed no significant correlation with antibody levels against N or RBD antigens (Supplementary Table S6). No correlation was observed between SARS-CoV-2 reactive antibodies and antibodies against the other infectious agents tested.

Supplementary Table S6. Correlation of SARS-CoV-2 antibody responses to antibody levels from other infectious agents.

Isotype	Antigen	CMV		EBV		HAVAb		Toxo		HBs	
		r	p-value	r	p-value	r	p-value	r	p-value	r	p-value
	N	0.24	0.062	0.29	0.026	-0.23	0.080	0.12	0.359	-0.05	0.719
Total (IgG/IgM/IgA)	S1	0.40	0.002	0.17	0.207	0.00	0.971	0.00	0.996	-0.09	0.499
	RBD	0.07	0.608	-0.05	0.701	-0.18	0.179	-0.04	0.754	-0.03	0.840
	N	0.19	0.156	0.32	0.013	-0.13	0.335	0.12	0.357	0.00	0.982
IgG	S1	0.43	0.001	0.24	0.066	0.14	0.303	0.00	0.995	-0.08	0.560
	RBD	0.06	0.666	0.22	0.101	0.05	0.699	0.00	0.973	-0.08	0.546
	N	0.33	0.011	0.11	0.402	0.09	0.491	-0.03	0.849	-0.07	0.578
IgA	S1	0.28	0.030	0.15	0.243	0.14	0.276	-0.01	0.941	-0.04	0.791
	RBD	0.17	0.192	0.11	0.421	-0.12	0.368	0.08	0.573	-0.11	0.415
	N	0.21	0.102	0.14	0.280	-0.18	0.173	0.01	0.928	0.03	0.794
IgM	S1	0.15	0.266	-0.01	0.926	-0.25	0.059	-0.02	0.869	-0.04	0.766
	RBD	0.11	0.410	-0.07	0.618	-0.20	0.137	-0.05	0.686	-0.01	0.946

S1.5 Receiver operating characteristic curve analysis

For each antibody ROC analysis is performed for the three (N, S1, RBD) antigens. The confidence intervals are calculated using Wilson's method. The AUC (area under the curve) of the ROC curves was calculated and used as a metric to evaluate the diagnostic performance of single antigen readouts (Supplementary Figure S4 & Supplementary Table S7).

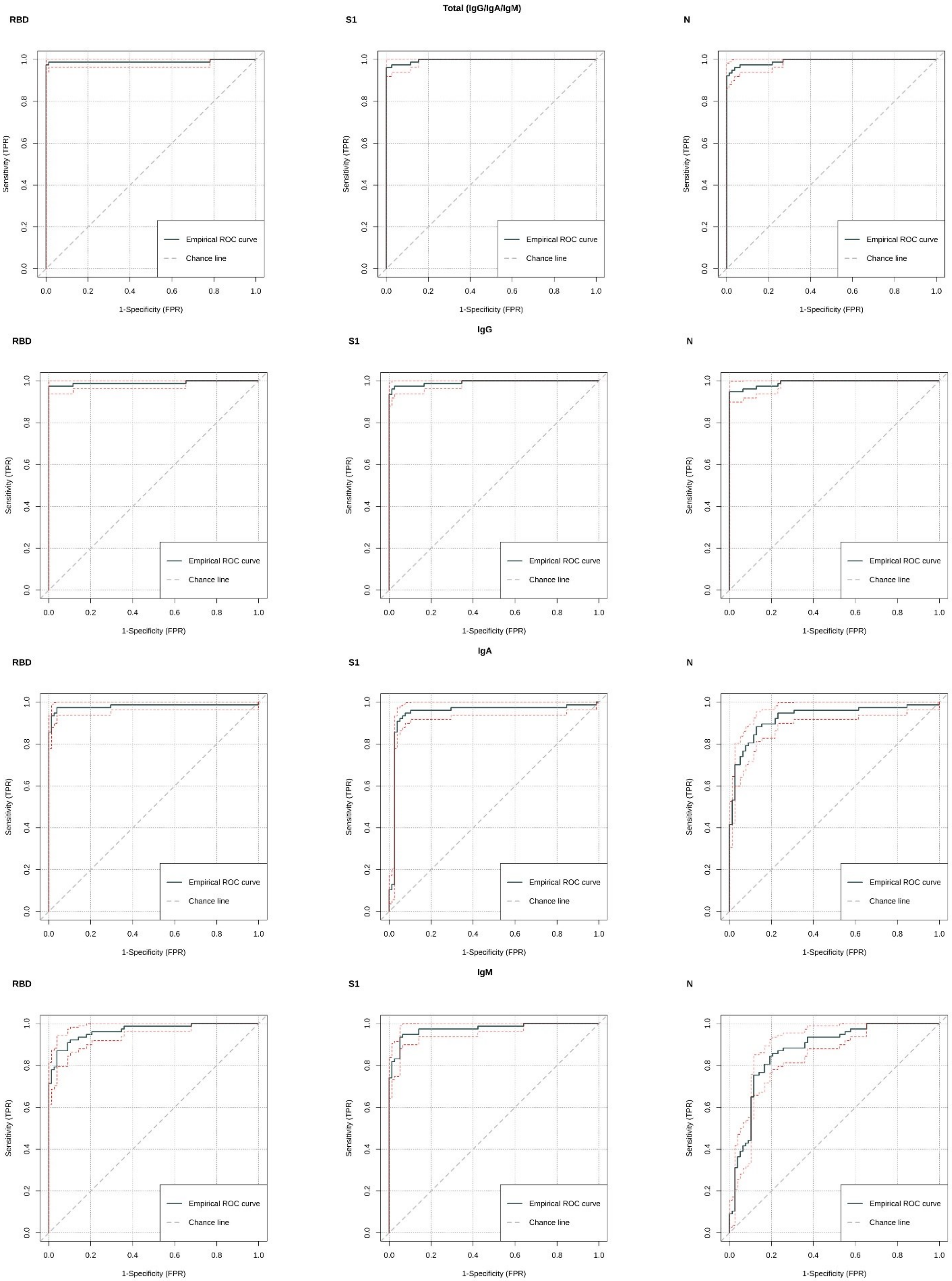

Total (IgG/IgA/IgM)
RBD
S1
N
IgG
RBD
S1
N
IgA
RBD
S1
N
IgM
RBD
S1
N
Sensitivity (TPR)
1-Specificity (FPR)
Empirical ROC curve
Chance line

Supplementary Figure S4. ROC curves for the 3 antigens and different antibody isotypes. Confidence intervals are demonstrated with red dotted lines.

Supplementary Table S7. Diagnostic accuracy and AUC of SARS-CoV-2 individual antigens.

**Total (IgG/IgM/IgA)**			**IgG**		**IgA**		**IgM**	
**Antigen**	**Accuracy** [95% CI]	**AUC** [95% CI]	**Accuracy** [95% CI]	**AUC** [95% CI]	**Accuracy** [95% CI]	**AUC** [95% CI]	**Accuracy** [95% CI]	**AUC** [95% CI]
N	0.961 [0.918,0.982]	0.992 [0.973, 1.000]	0.961 [0.918,0.982]	0.991 [0.980, 1.000]	0.755 [0.681,0.816]	0.928 [0.885, 0.971]	0.600 [0.521,0.674]	0.872 [0.815, 0.929]
S1	0.968 [0.927,0.986]	**0.996** [0.986, 1.000]	0.968 [0.927,0.986]	**0.993** [0.979, 1.000]	0.677 [0.600,0.746]	0.948 [0.911, 0.984]	**0.903** [0.845,0.940]	**0.975** [0.950, 1.000]
RBD	**0.981** [0.945,0.993]	0.990 [0.973, 1.000]	**0.981** [0.945,0.993]	0.990 [0.974, 1.000]	**0.955** [0.910,0.978]	**0.981** [0.959, 1.000]	0.884 [0.824,0.925]	0.966 [0.937, 0.996]

S1.6 Seroprevalence and agreement rates of single and multi-antigen rules in the population screening

Seroprevalence rates calculated using the best performing multi-antigen rules for different cut-off values are presented in Supplementary Figure S5. The agreement rates between all multi-antigen rules and between rules and commercial tests are presented in Supplementary Figures S6 & S7.

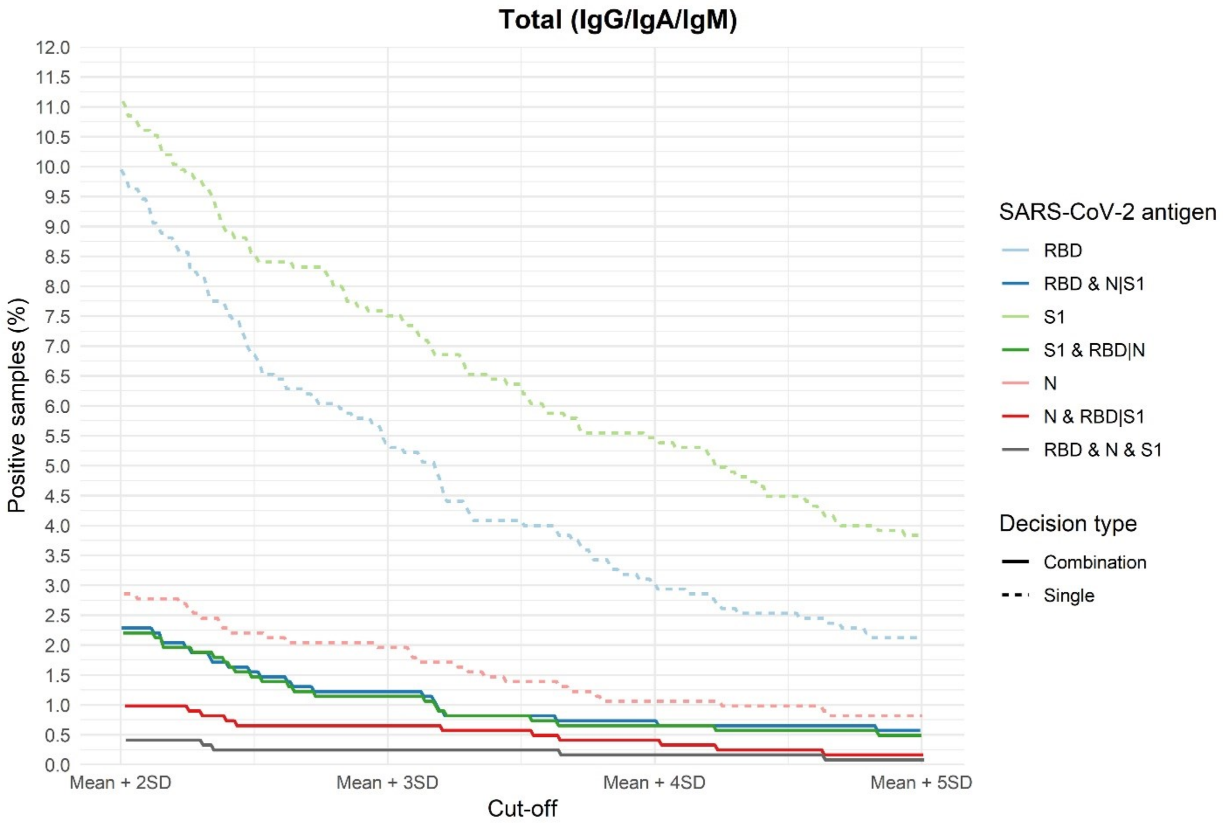

Supplementary Figure S5. Percentage of asymptomatic individuals (n=1,255) positive for total (IgG/IgA/IgM) SARS-CoV-2 antibodies using single antigen readouts and multi-antigen rules. Seroprevalence is calculated using different cut-off values from the distribution of negative samples used in the diagnostic performance analysis.

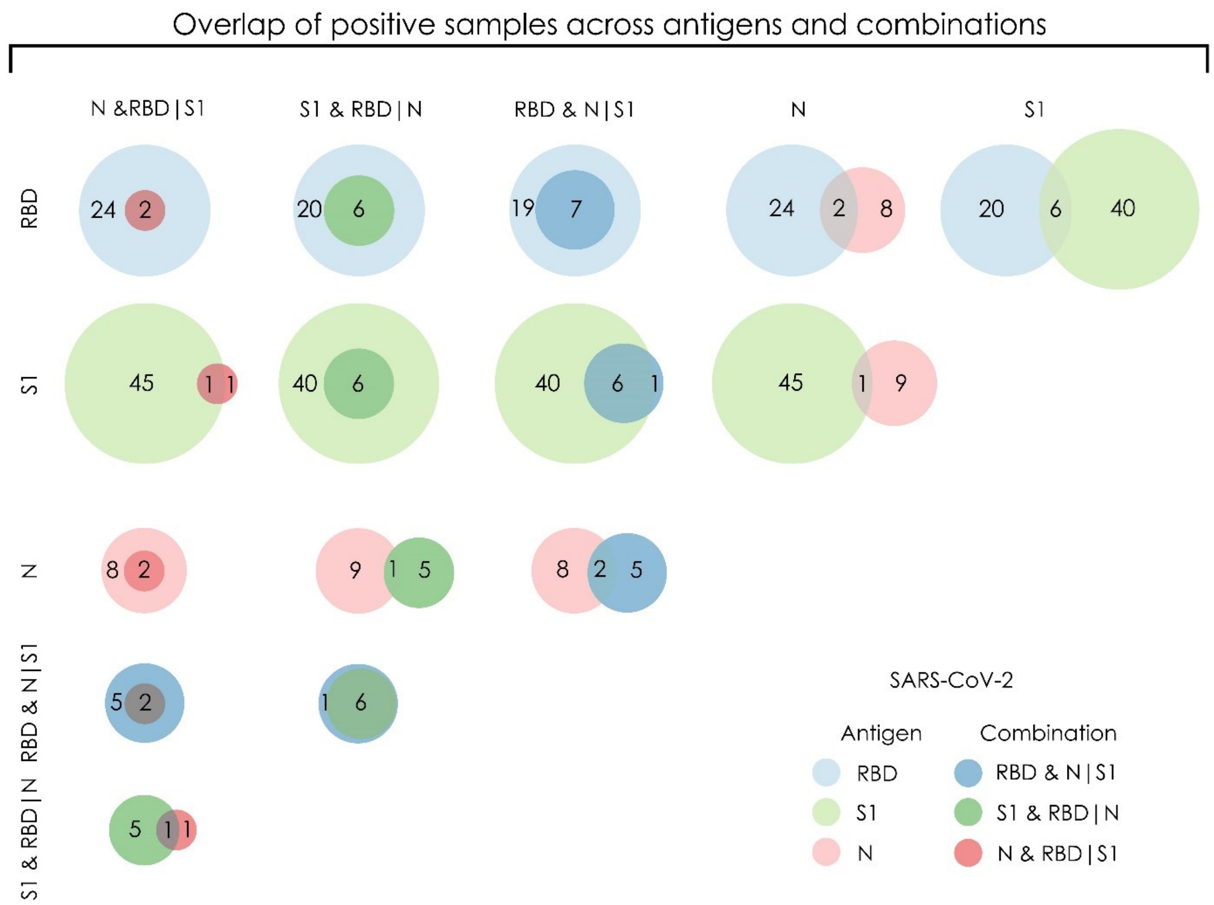

Supplementary Figure S6. Consensus between positive samples for total (IgG/IgA/IgM) antibodies using N, S1, RBD and respective multi-antigen rules in the population screen.

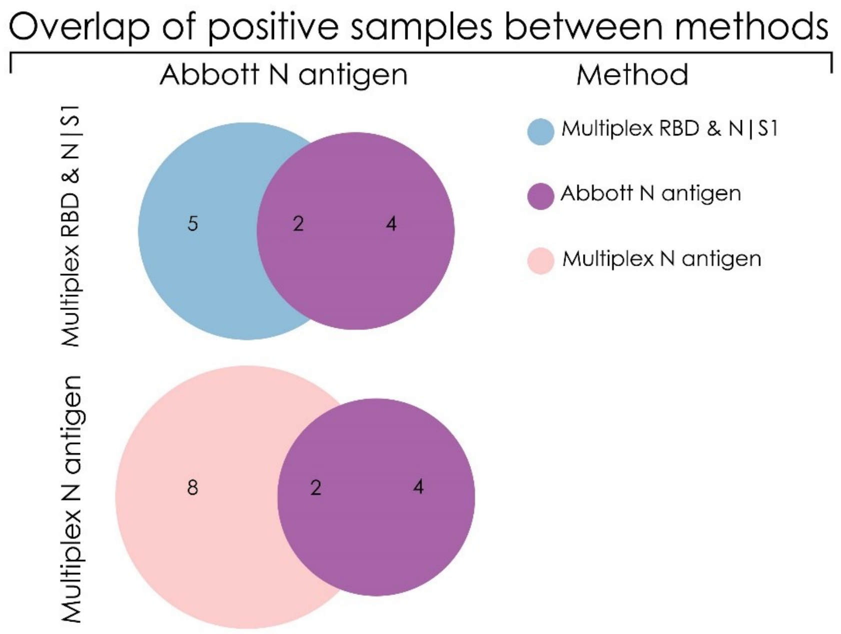

Supplementary Figure S7. Consensus between positive samples for total (IgG/IgA/IgM) antibodies against N, S1 and RBD and for IgG antibodies against N antigen (Abbott) in the population screen.

S.2 Supplementary methods

S.2.1 Clinical and donor samples

The list of all samples utilized in this study is presented in Supplementary Table S2.

S.2.2 Assay validation

Assay validation was performed in two separate subsets of matched clinical samples against two widely used, commercially available SARS-CoV-2 antibody tests developed by Euroimmun (Euroimmun Medizinische Labordiagnostika AG, Lubeck, Germany) and Abbott (Abbott Diagnostics, Illinois, USA), which detect IgG antibodies against S1 and N respectively (Supplementary Table S8).

Supplementary Table S8. Commercial SARS-CoV-2 serological assays and samples used for assay validation.

Commercial Assay	Antigen Tested	Total No of Samples	Sample Type	Sample Source
Euroimmun SARS-CoV-2 ELISA IgG	S1	60	Positive Serum (SARS-CoV-2 PCR-tested positive donors)*	Alexandra General Hospital, Athens
Abbott SARS-CoV-2 IgG	N	31	Positive Serum (SARS-CoV-2 PCR-tested positive donors)* (n=12)	University Hospital of Patras
			Serum from SARS CoV-2 positive or negative individuals (n=19)	University Hospital of Patras
